# Genomic adaptation to extreme climate conditions in beef cattle as a consequence of cross-breeding program

**DOI:** 10.1186/s12864-023-09235-2

**Published:** 2023-04-06

**Authors:** Rugang Tian, Hojjat Asadollahpour Nanaie, Xiao Wang, Baolige Dalai, Meng Zhao, Feng Wang, Hui Li, Ding Yang, Hao Zhang, Yuan Li, Tingyue Wang, Tu Luan, Jianghong Wu

**Affiliations:** 1 Inner Mongolia Academy of Agricultural & Animal Husbandry Sciences, Hohhot, 010031 China; 2 Key Laboratory of Animal Genetics, Breeding and Reproduction of Shaanxi Province, College of Animal Science and Technology, Northwest A&F University, Yangling, 712100 China; 3 Faculty of Biosciences, Norwegian University of Life Sciences, As, Norway; 4 College of Animal Science and Technology, Inner Mongolia Minzu University, Tongliao, China

**Keywords:** Cattle, Cold adaptation, Whole-genome sequence, *TRPM8*

## Abstract

**Background:**

Understanding the evolutionary forces related to climate changes that have been shaped genetic variation within species has long been a fundamental pursuit in biology. In this study, we generated whole-genome sequence (WGS) data from 65 cross-bred and 45 Mongolian cattle. Together with 62 whole-genome sequences from world-wide cattle populations, we estimated the genetic diversity and population genetic structure of cattle populations. In addition, we performed comparative population genomics analyses to explore the genetic basis underlying variation in the adaptation to cold climate and immune response in cross-bred cattle located in the cold region of China. To elucidate genomic signatures that underlie adaptation to cold climate, we performed three statistical measurements, fixation index (*F*ST), log_2_ nucleotide diversity (θπ ratio) and cross population composite likelihood ratio (XP-CLR), and further investigated the results to identify genomic regions under selection for cold adaptation and immune response-related traits.

**Results:**

By generating WGS data, we investigated the population genetic structure and phylogenetic relationship of studied cattle populations. The results revealed clustering of cattle groups in agreement with their geographic distribution. We detected noticeable genetic diversity between indigenous cattle ecotypes and commercial populations. Analysis of population structure demonstrated evidence of shared genetic ancestry between studied cross-bred population and both Red-Angus and Mongolian breeds. Among all studied cattle populations, the highest and lowest levels of linkage disequilibrium (LD) per Kb were detected in Holstein and Rashoki populations (ranged from ~ 0.54 to 0.73, respectively). Our search for potential genomic regions under selection in cross-bred cattle revealed several candidate genes related with immune response and cold shock protein on multiple chromosomes. We identified some adaptive introgression genes with greater than expected contributions from Mongolian ancestry into Molgolian x Red Angus composites such as *TRPM8*, *NMUR1*, *PRKAA2*, *SMTNL2* and *OXR1* that are involved in energy metabolism and metabolic homeostasis. In addition, we detected some candidate genes probably associated with immune response-related traits.

**Conclusion:**

The study identified candidate genes involved in responses to cold adaptation and immune response in cross-bred cattle, including new genes or gene pathways putatively involved in these adaptations. The identification of these genes may clarify the molecular basis underlying adaptation to extreme environmental climate and as such they might be used in cattle breeding programs to select more efficient breeds for cold climate regions.

**Supplementary Information:**

The online version contains supplementary material available at 10.1186/s12864-023-09235-2.

## Background

The worldwide demand for animal protein products (such as meat and milk) has greatly increased over the last decades and is expected to grow to about 50% until 2050, resulting in an increased need to improve the efficiency of livestock productivity and their management [1, 2]. Beef production, with annual over 70 million tons of meat, accounted as the largest supplier of red meat for human consumption [3]. China ranks third globally and first in Asia in terms of producing beef [4, 5]. In the last decade, with the rapid development of the Chinese economy, the amount of beef consumption has grown around 30%, ranking second in the world. Inner Mongolia of China with over 25% of the total area of grasslands and around one-fourth of its pasture region has a long history of exercising traditional nomadic pastoralism and remains an important bases of livestock production in the world [6–8]. Indigenous livestock of this region have become adapted to their local conditions, such as extreme environmental climate through natural selection. For example, Mongolian indigenous cattle have evolved to survive during tremendously cold winters and have reduced susceptibility to parasites, bacterial and viral infections [9, 10].

In recent decades, efforts have been made to improve the genetic potential of local cattle through crossbreeding with breeds introduced from other geographic regions. So far, various new crossbred populations have been developed with high production capacity and adaptability to different climates [11–13]. The new breeds, with their enhanced capacity for rapid growth and adaptation to climate changes, generally combine the favorable traits that characterized their purebred parental breeds through artificial and natural selection since the early 1970s. Among the world-wide commercial beef cattle, Red Angus is one of the most popular beef cattle breeds that has been widely used in crossbreeding programs to improve the carcass quality traits of local breeds. This breed has been separately registered from the black Angus cattle and is well known for its excellent carcass traits including marbling (intra-muscular fat) [14, 15]. As early as in 2000, by crossing this commercial breed with local populations that originated from Mongolian cattle (such as Chinese Steppes Red), a new cross-bred composite was generated with a variety of excellent characteristics, such as good meat quality, rapid growth and high adaptation ability to withstand extreme local climate conditions.

In cattle breeding, genome-wide sequencing has been a powerful way of identifying candidate genes related to body temperature [16] and economically important traits in both purebred and crossbreed populations [17–20]. In this study, we conducted a comprehensive population genomic analysis of 171 world-wide domestic modern cattle populations distributed across the world to investigate the phylogenetic relationships and identify key genetic variants associated with adaptation to extreme local climate in Mongolia region. Our findings will be helpful in selecting for cattle adapted to extreme environments, specifically in the development of cross-bred composite animals with tolerance for local climate conditions.

## Methods

### Sample collection and sequencing

**Fig. 1:**
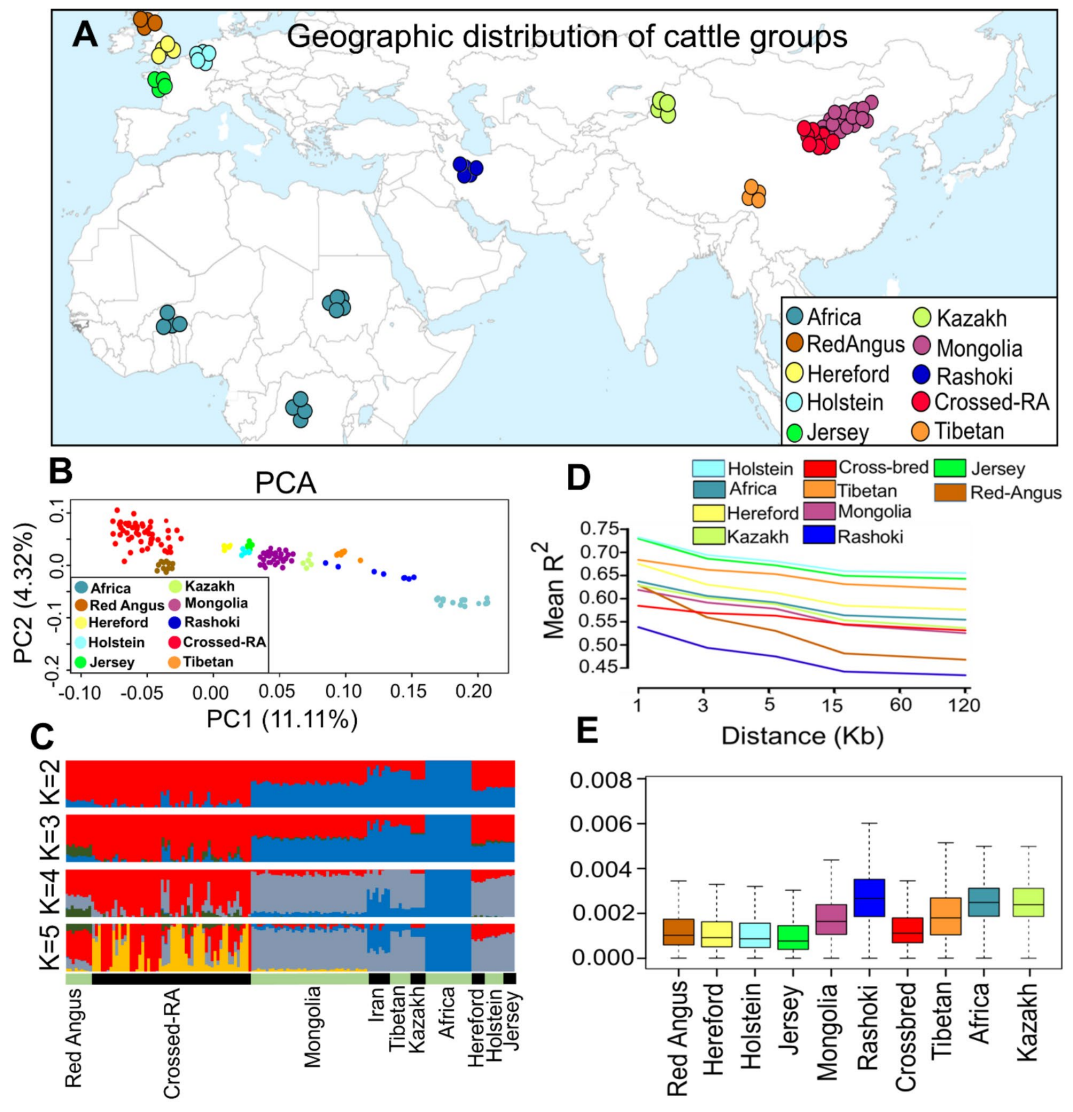
(A) Locations of cattle breeds for each geographic groups. Populations are colored to mirror their geographic origin. (B) PCA of cattle populations. (C) ADMIXTURE results for *k* = 2 to *k* = 5. (D) The decay of LD estimated as the squared correlation coefficient by pairwise physical distance in cattle populations and (E) Boxplots of nucleotide diversity, calculated in 50 kb sliding window with 20 kb increments across the genome

Historically, Red angus cattle were imported into China in the year of 1974, with no more than 50 animals. The majority of imported animals were female and semen was imported afterward. Since 2000, cross-breeding program (Mongolian x Red Angus) was started in order to produce animals with high performance capacities. In this study, we generated whole genome sequence (WGS) data from 65 cross-bred beef cattle (Mongolia × Red-Angus) and 45 Mongolian indigenous individuals. We further obtained a total of 61 complete genomes from world-wide cattle populations including; commercial (Red- Angus (n = 10), Hereford (n = 5), Holstein (n = 5) and Jersey (n = 5)) and native (Africa (n = 16), Rashoki (n = 8), Tibetan (n = 7), and Kazakh (n = 5)) cattle from the Sequence Read Archive (ncbi.nlm.nih.gov), resulting in a dataset of 171 cattle (Fig. 1A, Additional file 1: Table S1, Additional file 2: Fig. S1). We further obtained on Yak genome (*Bos grunniens*) as an outgroup for phylogeny analysis. Red Angus cattle were sampled from one farm at Xilingol in Inner Mongolia, and Mongolian cattle samples were collected from two farms (Tuoxian and Etuoke) in Inner Mongolia. Using the phenolchloroform method, genomic DNA was isolated from blood samples. The quality and concentration of extracted DNA were tested by agarose gel (2%) electrophoresis and NanoDrop spectrophotometer analysis, and then good-quality DNA samples were used for the subsequent whole genome resequencing. The short reads sequence data for all individuals were produced using the Illumina Hiseq 2500 platform, with a read length of 125 bp (Additional file 1: Table S1).

### Quality checking, alignments and SNP calling

The quality of the raw reads was explored and visualized by FastQC software (Version 0.4.2) (https://www.bioinformatics.babraham.ac.uk/projects/fastqc/). Adapter sequences and low level quality base pairs were trimmed by Trimmomatic software (version 0.36) [21]. Pair-end reads from the present study and published data that passed the filtering were aligned against the latest bovine reference genome assembly using Burrows Wheeler Aligner (BWA mem, V 0.7.16) [22]. The SAMtools software was used to discard un-mapped and non-unique reads, and for converting mapping results to the SAM (.sam) and BAM (.bam) formats [23]. The preprocessing of alignments, including removing potential PCR duplicates, was performed using Picard toolkit (http://broadinstitute.github.io/picard/). To improve the alignment accuracy, we then performed base quality score recalibration (BQSR) and local indel realignment using Genome Analysis Toolkit (GATK) tools [24]. Single-individual gVCFs were generated using the GATK HaplotypeCaller, and batches of 172 gVCFs were merged into a single gVCF with option CombineGVCFs. The GATK GenotypeGVCFs tool was then run to generate the joint genotyping of the gVCFs on all of the individuals together to create a raw SNP VCF. Subsequent filtering was further performed according to the GATK Best Practices recommendations. Final variants (~ 14.23 million SNPs) were filtered to be supported by a minimum mapping quality of 25 and a minimum genotype quality of 40. To exclude potential false-positive variant calls from the SNPs, we also used the “VariantFiltration”of GATK with the parameters “QD < 2.0, FS > 60.0, MQ < 40.0, MQRankSum < -12.5, ReadPosRankSum < -8.0 and SOR > 3.0”. In addition, all detected loci with more than two alleles and within clusters (> 3 SNP in a 10-bp window) were further filtered to avoid the potential sequencing errors [25]. After filtering out low-quality loci, the remained variants (around 12.36 million variants) were used for subsequent analyses.

### Population genetics analysis

We pruned our data set using PLINK v1.9 software (default settings) to minimize the non-independence of variations with options “–indep-pairwise 50 10 0.2” [26]. We then used the vcf2fq implemented in Samtools [23] to convert filtered VCF file into consensus FASTA file. A maximum likelihood (ML) phylogeny tree was constructed using FastTree2 software (version 2.1.11) [27]. The topological structure was visualized with online tool iTOL (V6; https://itol.embl.de/). The principal component analysis (PCA) was performed using smartpca of the EIGENSOFT package with the “lsqproject” and “autoshrink” options [28]. To further understand the degree of Admixture in the population, we used the Bayesian clustering method implemented in ADMIXTURE software, with number of ancestral populations (*K*) ranging from 2 to 5 and 10,000 iterations for each run [29]. Additionally, to study fine-grained population genetic structure across all cattle groups, haplotype sharing patterns were investigated using the algorithm implemented in ChromoPainter/fineSTRUCTURE softwares [30]. Individual inbreeding coefficients (F) were estimated using the PLINK “het” function [26]. To evaluate the recent and historical effective population size (Ne), linkage disequilibrium (LD) decay rates between adjacent SNPs across the whole genome were calculated for each cattle population.

### Identification of selective sweeps and adaptive introgression regions related to local adaptation in cross-bred cattle

To identify genomic regions under selection, three different statistical approaches including fixation index (*F*ST), log_2_ nucleotide diversity (θπ ratio), and cross population composite likelihood ratio (XP-CLR) methods were calculated. For both *F*ST and Pi tests, we set the window size of 50 kb and the sliding step of 25 kb via vcftools (-‐window‐pi 50,000 ‐‐window‐pi‐step 25,000) [31]. For the XP-CLR test statistic, we used the following options: grid size 10 K, sliding windows of 0.1 cM, maximum number of SNPs within each window as 300, and correlation value for 2 SNPs weighted with a cutoff of 0.99. To identify signals of introgression between cross-bred animals and Mongolian cattle, a sliding-window analysis was conducted using Dinvestigate program (*f*d statistic) to explore whether the admixture signal was confined to specific contigs [32]. We highlighted introgression candidate genes as trios with significant values (Bonferroni-corrected p-value < 0.001) between studies populations.

## Results

### Dataset, population genetic structure and admixture

Together with the publicly available whole genome data (n = 62), we generated complete genome sequencing data from 65 cross-bred beef cattle (Mongolia × Red-Angus) and 45 Mongolian indigenous individuals (Fig. 1A, Additional file 1: Table S1, Additional file 2: Fig. S1). After quality filtering, the average sequence depth was ~ 9.8 per each sample (covering from ~ 4.0X to ~ 27.0X), which is an ideal practical depth for discovering accurate variants [33] (Additional file 1: Table S1). To identify genetic relationship between all studied groups, we first reconstructed a rooted ML tree. Based on this tree all commercial cattle breeds were clustered close together and cross-bred animals were formed between Red-Angus and Chinese Kazakh and Mongolian groups (Additional file 2: Fig. S2). We noticed that the majority of Tibetan cattle were located close to the Yak genome, which is in line with previous studies [34]. The same population affinities were also recovered in PCA analyses. The PC1 and PC2 accounted for 11.11% and 4.32% of the total genotypic variance, respectively. The first component was driven by difference between commercial groups and indigenous cattle (Fig. 1B). Commercial cattle groups could further be classified into two major groups, Red-Angus with its cross-bred samples and other cattle populations (Hereford, Holstein and Jersey). ADMIXTURE (from *K*2 to *K*5) analysis also recapitulated these findings. Ancestral proportions at *K* = 2 indicated that Red-Angus cattle and cross-bred animals were mainly assigned to the same cluster, while African individuals were separated from other groups (Fig. 1C). When *K* = 4, with the lowest CV error (Additional file 2: Fig. S3), we further observed that cross-bred cattle derive from genetic admixture between Mongolia and Red-Angus cattle (Fig. 1C). Our LD decay results show that the r^2^ scores were the highest at marker pairs distance of 1 Kb (ranged from ~ 0.54 to 0.73, for Rashoki and Holstein cattle, respectively) with a gradual decline with increasing physical distance (up to 50 Kb) and then stable trend (> 50 Kb) (Fig. 1D). The results further show that from marker pairs distance of 1 Kb to ≤ 15 Kb, the decay of LD was more rapid in both Red-Angus and Rashoki groups than other cattle populations, reaching an average r^2^ values from 0.64 to 0.52 and 0.54 to 0.45, respectively (Fig. 1D). Focusing on all studied indigenous groups, lower r^2^ values across all genomic distances were found for Rashoki cattle that dwelled in northern parts of Iran country. We next estimated the nucleotide diversity within each cattle population. The results revealed that all commercial cattle have lower genetic diversity than other indigenous breeds, and among native groups, both Rashoki samples from Iran and indigenous cattle from Africa show higher genetic diversity than other populations (Fig. 1E).

Furthermore, we used ChromoPainter together with the finestructure clustering to better characterise the genetic relationship of all studied groups. The obtained findings are summarized into a “co-ancestry matrix”. Each column and each row reveals the result of expected coancestry between each sample and other animals in the whole genome dataset (Additional file 2: Fig. S4). In agreement with admixture estimates (*K* = 2), the results of painting algorithm show African samples have a lower genetic similarity with other world-wide cattle populations.

**Fig. 2:**
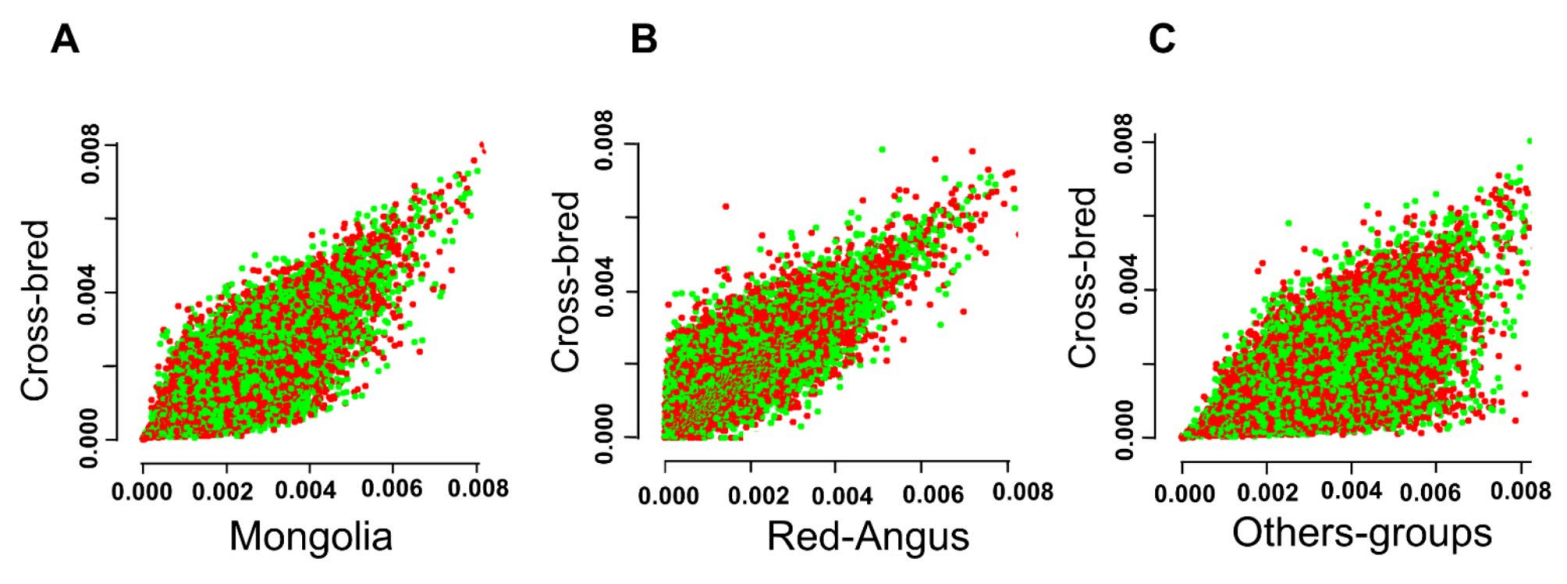
Correlation of nucleotide diversity (θπ) (50-kb non-overlapping window) between cross-bred group (green) and different cattle groups; (A) Mongolian cattle, (B) Red-Angus breed and (C) other populations (orange)

The analysis of patterns of genetic divergence, as a function of θπ, between gene pools of different cattle populations revealed a strong correlation between cross-bred animals and both Red-Angus and Mongolian cattle, which is clearly due to their shared genetic ancestry with these two cattle groups (Fig. 2).

Additionally, we estimated inbreeding coefficients per each sample (ranged from ~ 0.01 to 0.60) and each cattle group (ranged from 0.05 to 0.55 for Rashoki and Holstein groups, respectively) (Additional file 2: Fig. S5). Among the indigenous cattle, the highest and lowest levels of average inbreeding coefficient were 0.22 and 0.05 for African and Rashoki groups, respectively. The results collectively show that native cattle populations had lower inbreeding coefficient than commercial groups.

### Signatures of adaptive introgression in cross-bred cattle

**Fig. 3:**
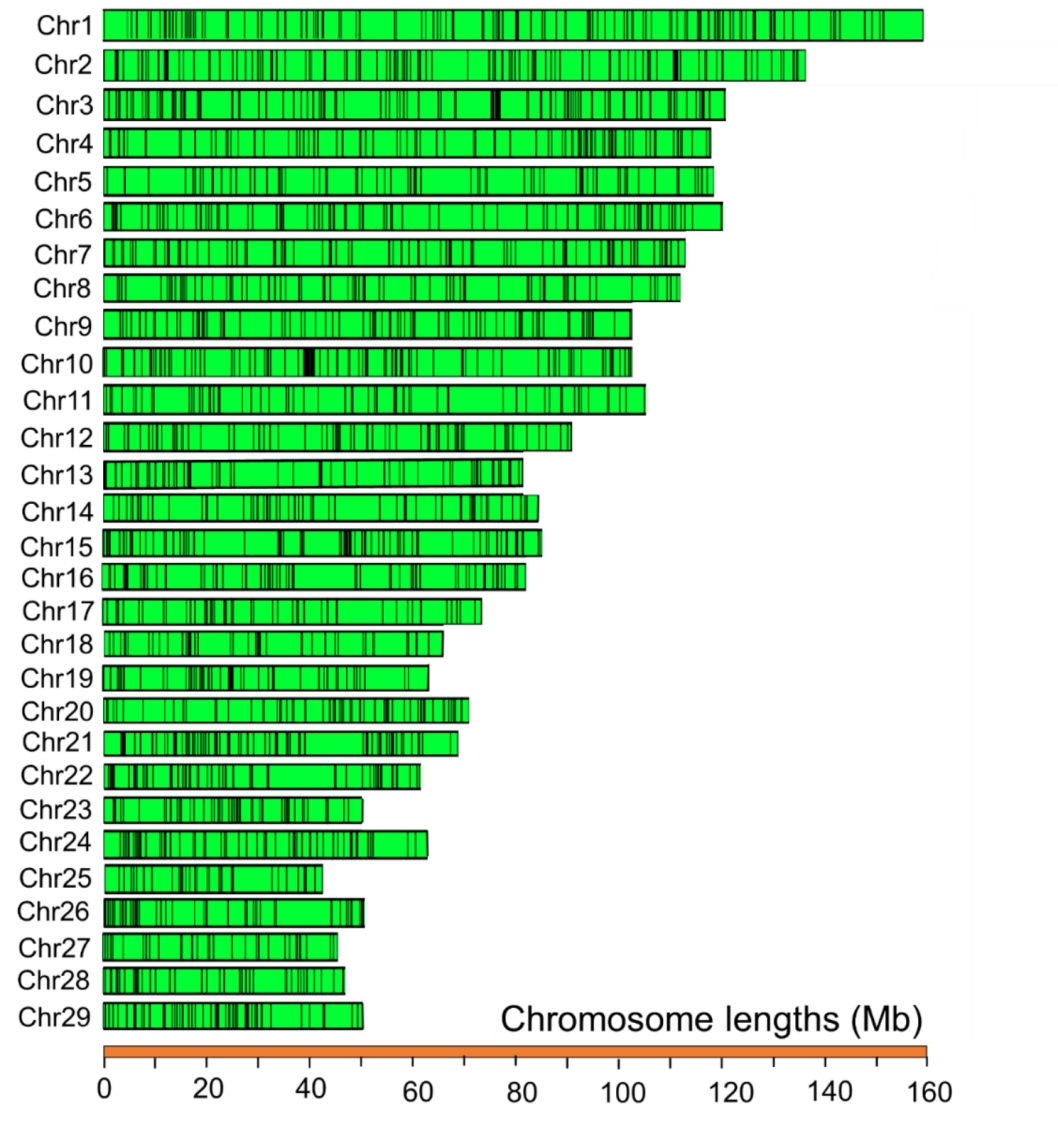
Genome-wild introgressions (black lines) from Mongolian cattle into cross-bred population

Previous genomic studies have confirmed that crossbreeding between different populations or geographically differentiated subspecies can increase genetic diversity and produce novel allelic combinations by new phenotypic traits [35, 36]. The adaptation of mammals in cold climate habitats may have effect on their phenotypic features, such as increased blood pressure, low serum lipid levels, as well as increased basal metabolic rate [37–39]. In this study, to identify adaptive introgression regions from Mongolian cattle into the cross-bred population, we performed *f*d statistic [32]. Selecting the top 1% of the introgressed regions, we detected several genomic coordinates (919 genes) as high-confidence introgressed segments (Fig. 3, Additional file 1: Table S2). The size of introgressed regions varies from several hundreds bp to more than 280 kb base pairs (ranging from 1.266 to 28.333 Kb). We then to explore the genetic architecture of cross-bred population and their adaptive evolution to cold climate conditions, compared their genome sequences with those from Red-Angus cattle, which have been previously imported to China.

**Fig. 4:**
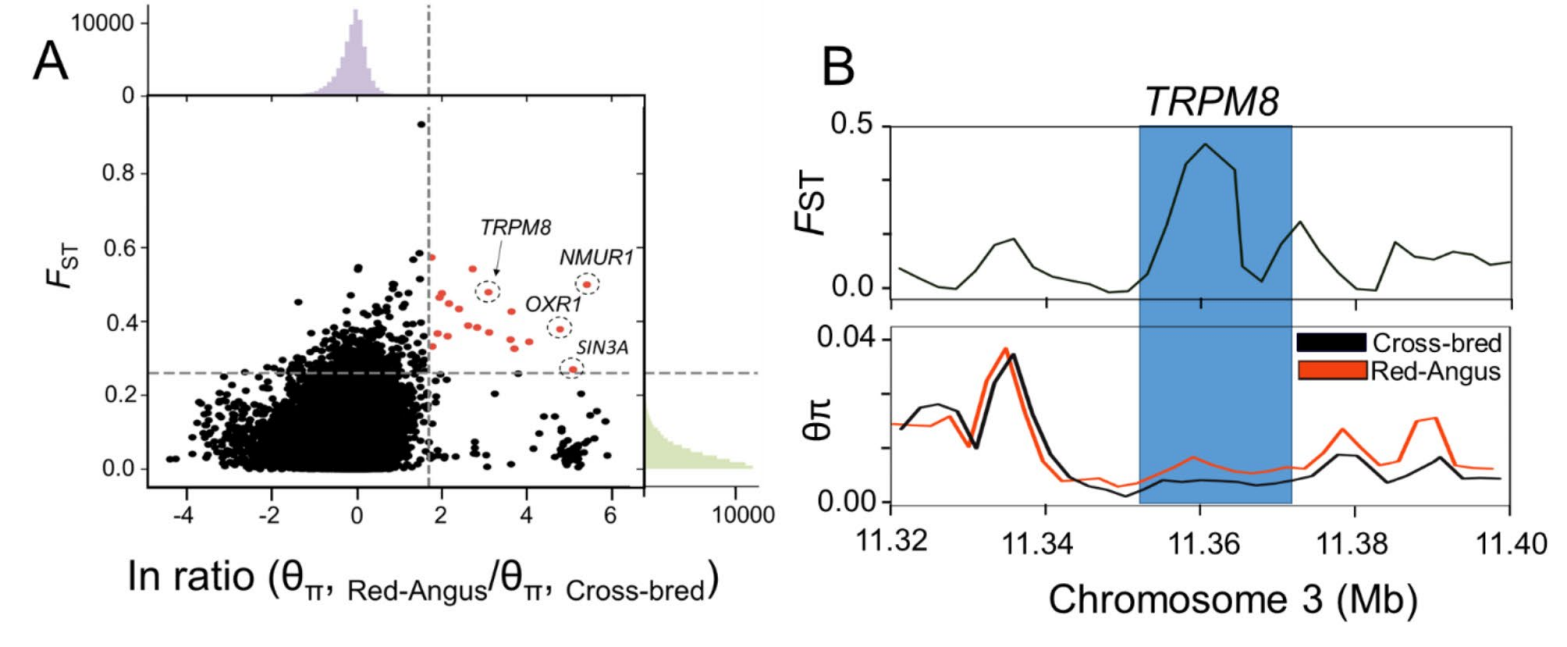
**Genomic regions with selection signals in cross-bred cattle group.** (A) Distribution of the pairwise fixation index (*F*ST) (y axis) and θπ ln ratio (x axis) between Red-Angus cattle and cross-bred group. (B) Selection signals around *TRPM8*. *F*ST based on SNPs is shown as a line using a nonoverlapping 10-kb sliding window

The results of comparative genomic analysis revealed a total of 475 and 394 protein-coding genes in windows with high *F*ST (1% cutoff) and log2 θπ ratio values (1% cutoff), respectively (Fig. 4, Additional file 1: Tables S3, S4). Among the strong selective signals (by both Pi and *F*ST approaches), we localized some candidate genes that were overlapped with those genes that were detected by *f*d statistics (Table 1). The identified genes are involved in variety of adaptation traits such as climate adaptations (e.g., *TRPM8*, *SMTNL2* and *OXR1*), energy metabolism (e.g., *NMUR1* and *PRKAA2*), and resistance to parasites (e.g., *SIN3A* and *PLCB4*). The overlap of candidate genes between introgression and selective sweep regions may suggest their role in adaptive mechanisms in cross-bred animals. Gene set enrichment analysis (GSEA) and KEGG mapping further identified some significantly enriched pathways (FDR %1) related with cold tolerant and energy metabolisms such as “Response to cold” (GO:0009409), “Thermoception” (GO:0050955), “Sensory perception of temperature stimulus” (GO:0050951), “cAMP metabolic process” (GO:0046058) and “cGMP metabolic process” (GO:0046068) (Additional file 1: Tables S5, S6). Based on the comparison of allele frequency spectrum, we additionally applied an XP-CLR test to identify genomic regions under selection. A total number of 362 candidate genes were detected in the top percentiles of approach (1% cutoff) (Additional file 2: Fig. S6; Additional file 1: Table S7). The functional enrichment analysis results on these candidate genes again highlighted the involvement of gene pathways related with cold tolerant and energy metabolisms such as “Thermoception” (GO:0050955), “Response to temperature stimulus” (GO:0009266), and “Detection of temperature stimulus” (GO:0009581) (Additional file 1: Table S8).

**Table 1:** Overlapped genes identified by signature selection methods (*F*ST, log2 θπ ratio and XP-CLR) and *fd* statistics, which are involved in adaptation traits to climate change in cross-bred cattle

Gene	Chr.^*^	Ensembl ID	Summary of gene function
*NMUR1*	2	ENSBTAG00000017684	Energy expenditure & Adaptation to climate change [56–58]
*TRPM8*	3	ENSBTAG00000014652	Adaptation to climate change [51–55]
*PRKAA2*	3	ENSBTAG00000018747	Energy metabolism [61, 62]
*SMTNL2*	19	ENSBTAG00000020605	Adaptation to climate change [63]
*SIN3A*	21	ENSBTAG00000009985	Immune response [64, 65]
*OXR1*	14	ENSBTAG00000008285	Thermoregulatory mechanism [66]
*PLCB4*	13	ENSBTAG00000013116	Immune response [59, 60]

*Chromosome

## Discussion

### Genetic diversity and population structure

In the present study, WGS data were used to analyze patterns of genetic variation and phylogenetic relationships of world-wide cattle populations. This information is essential towards improvement of genetic potential of local cattle populations. In agreement with previous studies [20, 40], phylogeny analysis showed clear separation between commercial cattle groups and indigenous populations. However, all samples from African cattle were clearly distinguishable from the other native breeds, Kazakh and Mongolian groups were clustered close together in either the phylogenetic tree and PCA, indicating the possibility of shared genetic components between these two Northwest Chinese breeds [34]. The distance patterns between studied groups was also observed by PCA and Admixture analysis. The cluster results indicated that cross-bred individuals had a close relationship with both Red-Angus and Mongolian cattle breeds, which is in accordance with their breeding history involving crossbreeding and selection. Furthermore, results from Bayesian-model clustering analysis showed high amount of shared genetic admixture between cross-bred cattle and Red-Angus breed, rather than Mongolian cattle, suggesting local farmers have been focused extensively on both growth rate and meat production traits in their crossbreeding programs. Estimating the patterns of genetic divergence further showed a strong correlation of nucleotide diversity between Red-Angus cattle and Cross-bred animals (Fig. 2).

Consistent with most previous studies, we observed that the commercial cattle populations harbor lower levels of genetic diversity, as compared with the indigenous groups, which could be the result of intensive artificial selection over generations [20, 34, 40]. Furthermore, we found that Jersey cattle had the lowest genetic diversity which is in line with the previous reports that have pointed out this cattle breed has experienced extensive inbreeding depression than other dairy cattle populations [41]. The relatively high level of nucleotide diversity in Rashoki cattle may reflect weaker, targeted, and shorter selection history in this populations. The pattern of LD decay can provide valuable insights into population history, including breeding systems and evolution of a population [42–44]. In this study, the LD values among SNPs decreased with increasing physical distance, which is in accordance with studies on different livestock species [42, 45]. The relatively low levels of LD in the entire genome of cross-bred animals are probably the result of past admixture events between Red-Angus and Mongolian cattle that have been experienced due to genetic improvement of local cattle breeds for meat production traits. We notice that as the genomic distance increased, all indigenous cattle groups followed the same pattern of decrease in LD, however more rapid decrease in LD over increasing genetic distance were identified in both Red-Angus and Rashoki, which may be due to differences in effective population size of these two cattle breeds.

### The adaptation of cross-bred cattle to environmental stresses

Although climate has a profound effect on the survival of organisms, our knowledge about the genomic mechanisms of cattle adaptation to extreme local climate (such as cold adaptation) is limited. Genomic studies have confirmed that the cross-bred populations may have the advantage of adaptability to both climate change and diseases resistance [46, 47]. Red-Angus has been derived from the Aberdeen Angus and is becoming an increasingly popular component of the agricultural sector. This breed is widely used in crossbreeding programs in local areas and has been noted for its high quality carcass traits [48]. In recent times, by crossing this breed with local cattle in Mongolia, a new synthetic cross-bred population with excellent tolerance to cold stresses has been generated.

In this study, by performing whole-genome selection scans between cross-bred animals and Red-Angus cattle, several genomic regions were found to be in the high-confidence selection regions (highest 1% of *F*ST, XP-CLR and log2 θπ ratio values) (Additional file 1: Tables S3, S4 and S7). In addition, by overlapping these results with those obtained from the introgression analysis, we identified some candidate genes that are related to environmental adaptation and metabolic homeostasis (such as; *TRPM8, NMUR1, OXR1, PRKAA2* and *SMTNL2)*, as well as immune response (e.g. *PLCB4* and *SIN3A*) (Table 1). The *TRPM8* gene was detected as a candidate gene in a selective sweep region located on bovine chromosome (BTA) 3, which consistently shows high signal values in *F*ST and XP-CLR analyses as well as the log2 θπ ratio(top 1% cutoff) (Fig. 4B, Additional file 2: Fig. S6). This gene encodes an ion channel that plays a critical role in mammalian thermosensation [49, 50]. Previous studies have suggested that *TRPM8* gene is activated by cold and cooling compounds, which makes it an important candidate as the molecular mediator of cold adaptation. Genetic variation of this gene has been reported in different species [51–53]. It has been demonstrated that the proteins encoded by this gene in brown adipocytes are involved in the regulation of body temperature, and are necessary for the survival of newborns and hibernating mammals [54]. Furthermore, it has been documented that the absence of *TRPM8* gene in some fish species can remarkably facilitate their colonization in a large range of climate zones [55]. *NMUR1* gene (also called *GPR66/FM-3*) is located on BTA 2 (119.283-119.294 Mb) and belongs to the G-protein-coupled receptors. Considering the important function of this gene in several important physiological functions such as energy expenditure, stress responses, feeding behavior and circadian rhythm, this gene is an important candidate for adaptation to climate change in cross-bred animals [56–58]. *PLCB4*, a phospholipase C beta 4, is a key player in different cellular processes such as neural signaling, cell growth and synaptic plasticity [59]. It has been reported that this gene is involved in regulating immune defense, energy metabolism and oxidative stress response in several species. A more recent study has also suggested that *PLCB4 *gene may related with immune response and environmental temperature in native chicken ecotypes [60].

## Conclusion

By applying whole-genome sequencing analysis, we were able to highlight candidate genes involved in responses to cold adaptation and immune response, including new genes such as *TRPM8*, *NMUR1*, *PRKAA2*, *SMTNL2* and *OXR1* or gene pathways (e.g. Response to cold, Thermoception and Sensory perception of temperature stimulus) putatively involved in these adaptations. These findings can therefore inform targeted genomic in vivo, and in vitro studies to further explore hypotheses arising from our study, and to investigate underlying genomic mechanisms.

## Electronic supplementary material

Below is the link to the electronic supplementary material.

**Additional file 1**

**Additional file 2**

## Data Availability

The datasets generated and/or analysed during the current study are available at NCBI SRA Database with accession code: PRJNA896136 (https://www.ncbi.nlm.nih.gov/).

## List of abbreviations

GATK: Genome Analysis Toolkit
VCF: Variant Calling File
BWA: Burrows-Wheeler Aligner
*F*_ST_: Fixation index
LD: linkage disequilibrium
GO: Gene ontology
θπ: nucleotide diversity
PCA: principal component analysis
ROH: Runs of homozygosity
SNP: single-nucleotide polymorphism
GCTA: genome-wide complex trait analysis
