# Correction: Genomic adaptation to extreme climate conditions in beef cattle as a consequence of cross-breeding program

**DOI:** 10.1186/s12864-024-11042-2

**Published:** 2024-11-20

**Authors:** Rugang Tian, Hojjat Asadollahpour Nanaie, Xiao Wang, Baolige Dalai, Meng Zhao, Feng Wang, Hui Li, Ding Yang, Hao Zhang, Yuan Li, Tingyue Wang, Tu Luan, Jianghong Wu

**Affiliations:** 1 Inner Mongolia Academy of Agricultural & Animal Husbandry Sciences, Hohhot, 010031 China; 2 Key Laboratory of Animal Genetics, Breeding and Reproduction of Shaanxi Province, College of Animal Science and Technology, Northwest A&F University, Yangling, 712100 China; 3 Faculty of Biosciences, Norwegian University of Life Sciences, As, Norway; 4 College of Animal Science and Technology, Inner Mongolia Minzu University, Tongliao, China

**Correction:** *BMC Genomics* **24, 186 (2023)**

10.1186/s12864-023-09235-2

Following publication of the original article it was reported that there was a typographical error in the name of Feng Wang.

It was incorrectly given as: Fenf Wang.

The original article has been corrected.

