# Supplementary material for: Genomic adaptation to extreme climate conditions in beef cattle as a consequence of cross-breeding program
Source: BMC Genomics. 2023 Apr 6;24:186. doi: 10.1186/s12864-023-09235-2 (PMC10080750; doi:10.1186/s12864-023-09235-2)

**Genomic adaptation to extreme climate conditions in beef cattle as a consequence of cross-breeding program**

Rugang Tian^1*†^, Hojjat Asadollahpour Nanaie^1,2†^, Xiao Wang^1^, Baolige Dalai^1^, Meng Zhao^1^, Fenf Wang^1^, Hui Li^1^，Ding Yang^1^, Hao Zhang^1^, Yuan Li^1^, Tingyue Wang^1^, Tu Luan^3^, Jianghong Wu^4*^

^1^Inner Mongolia Academy of Agricultural & Animal Husbandry Sciences, Hohhot, 010031, China

^2^ Key Laboratory of Animal Genetics, Breeding and Reproduction of Shaanxi Province, College of Animal Science and Technology, Northwest A&F University, Yangling 712100, China

^3^Faculty of Biosciences, Norwegian University of Life Sciences

^4^ College of Animal Science and Technology, Inner Mongolia Minzu University

†These authors contributed equally to this work.

*Corresponding author:

Prof. Rugang Tian

E-mail: [tiannky@163.com](mailto:tiannky@163.com)

Phone: +86 15124784023

Dr. Jianghong Wu

E-mail: wujianghonglong@126.com

Phone: +86 18947916076

**Fig. S1**: Cattle breeds used in this study. The figure was designed by the first author.


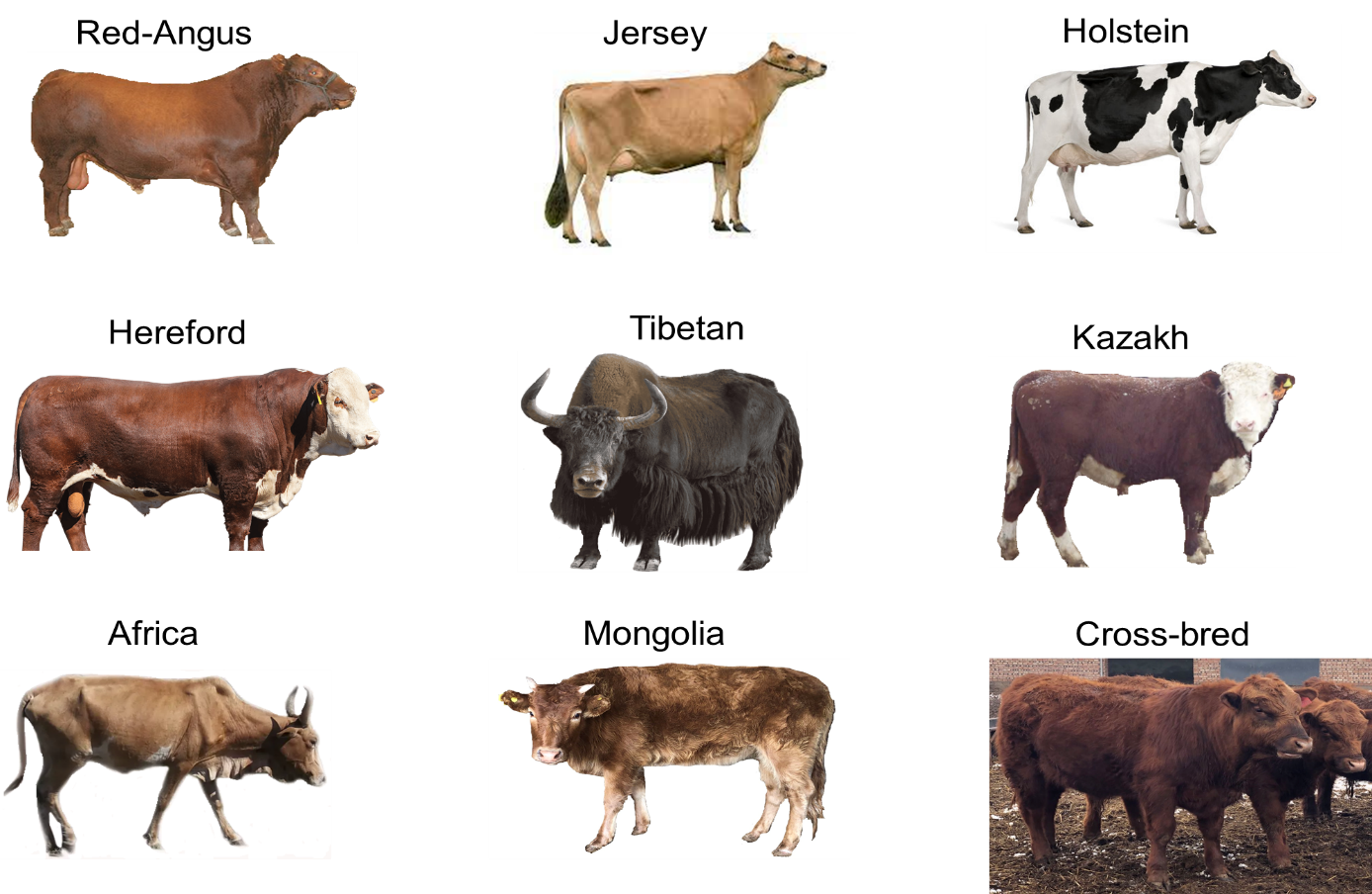


**Fig. S2.** A phylogenic tree from the genome sequences used in this study. The branches are colored following the same color code used in Fig.1A.

**
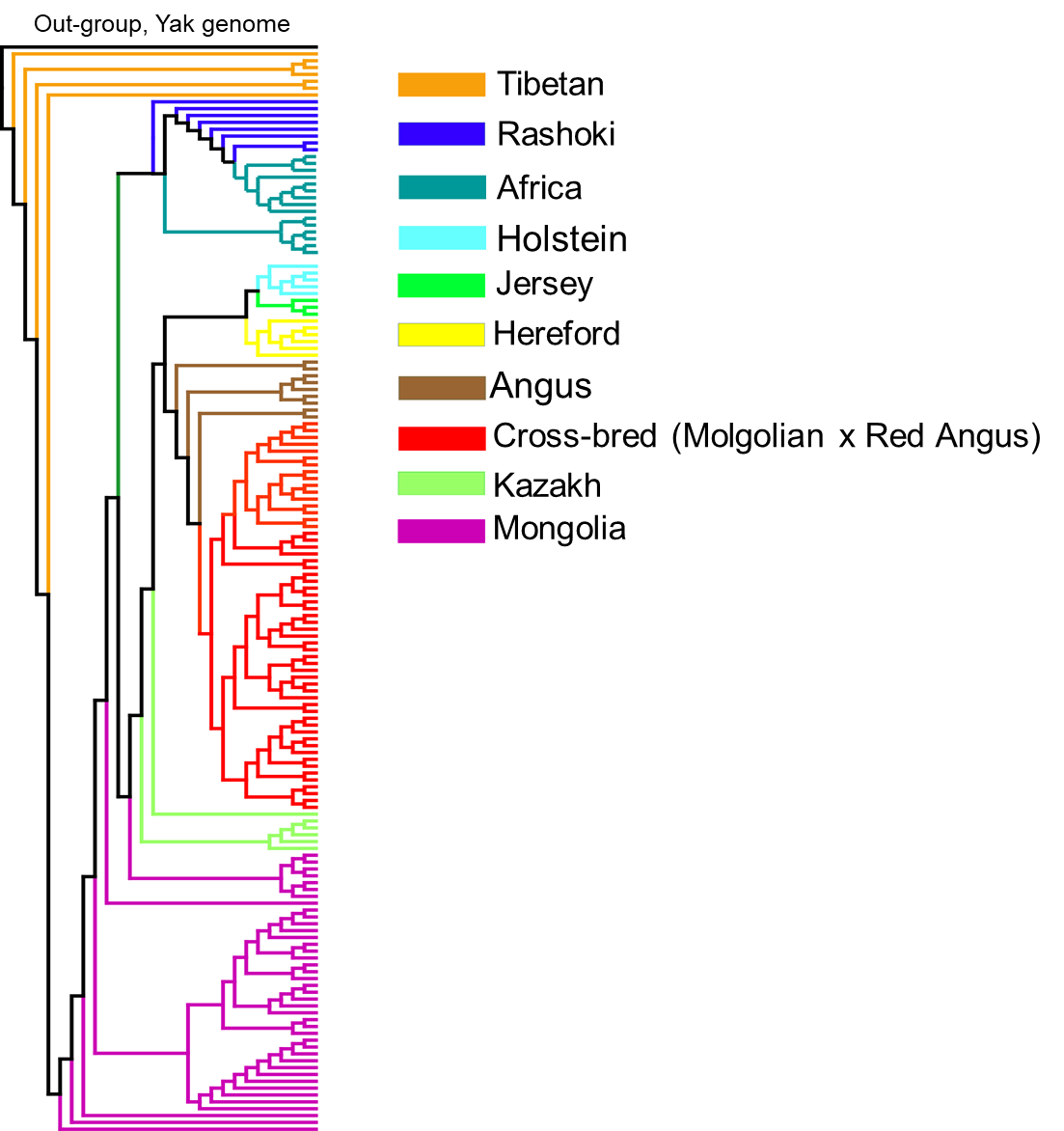
**

**Fig. S3**: Cross validation error (CV) plot from ADMIXTURE.


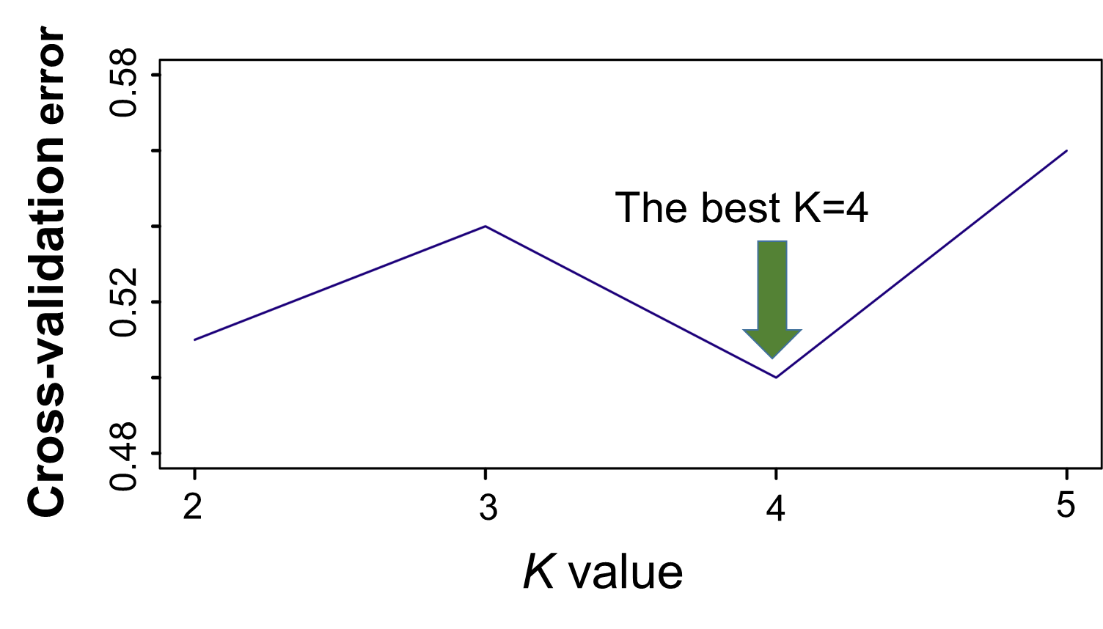


**Fig. S4.** The heat map of ChromoPainter’s coancestry matrix. The African individuals (red box) show lower haplotype sharing with other world-wide cattle groups.


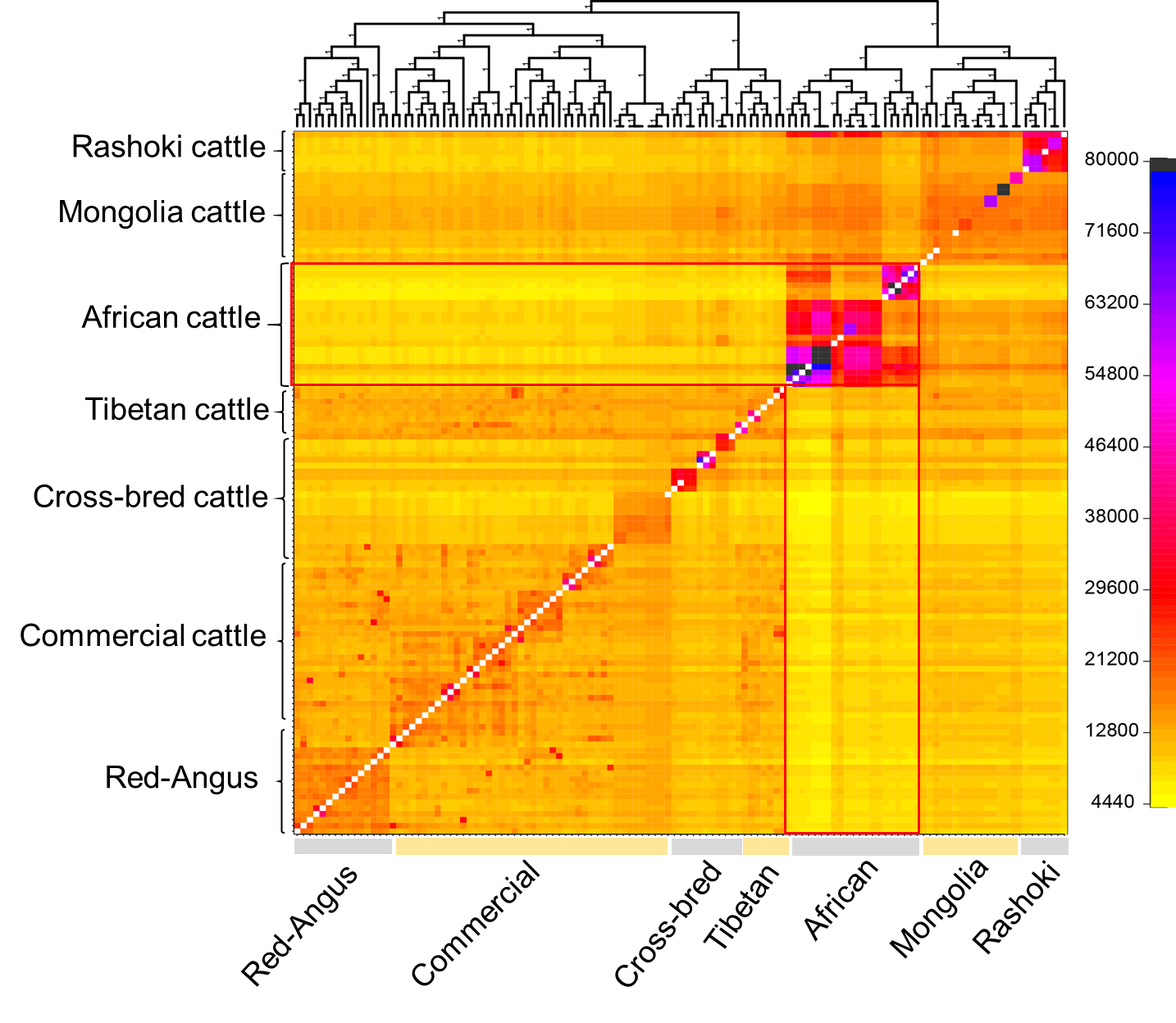


**Fig. S5.** Inbreeding coefficient (F) for different cattle populations in this study.

**
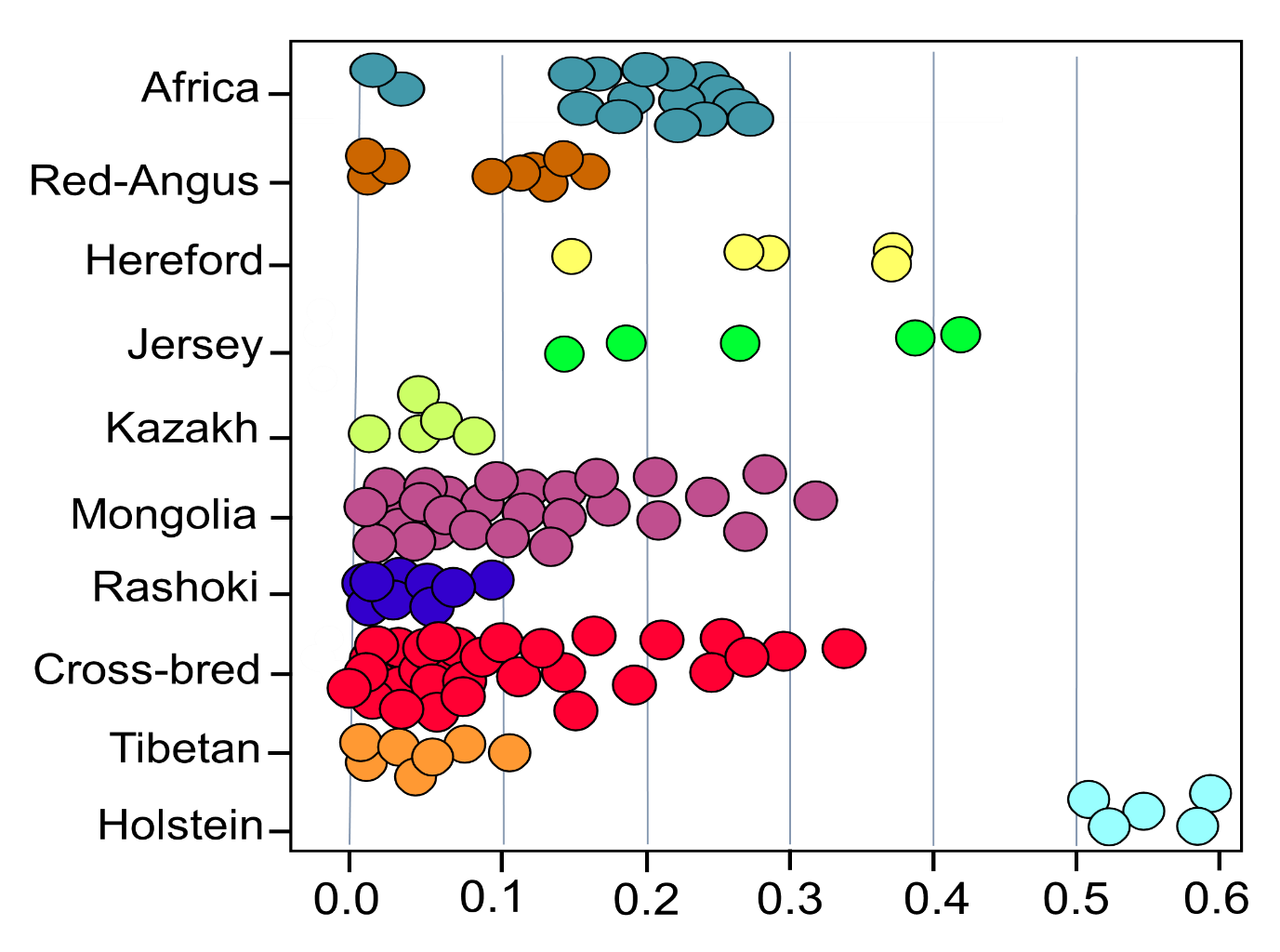
**

**Fig. S6**. Genomic landscape of population differentiation by XP-CLR statistic.


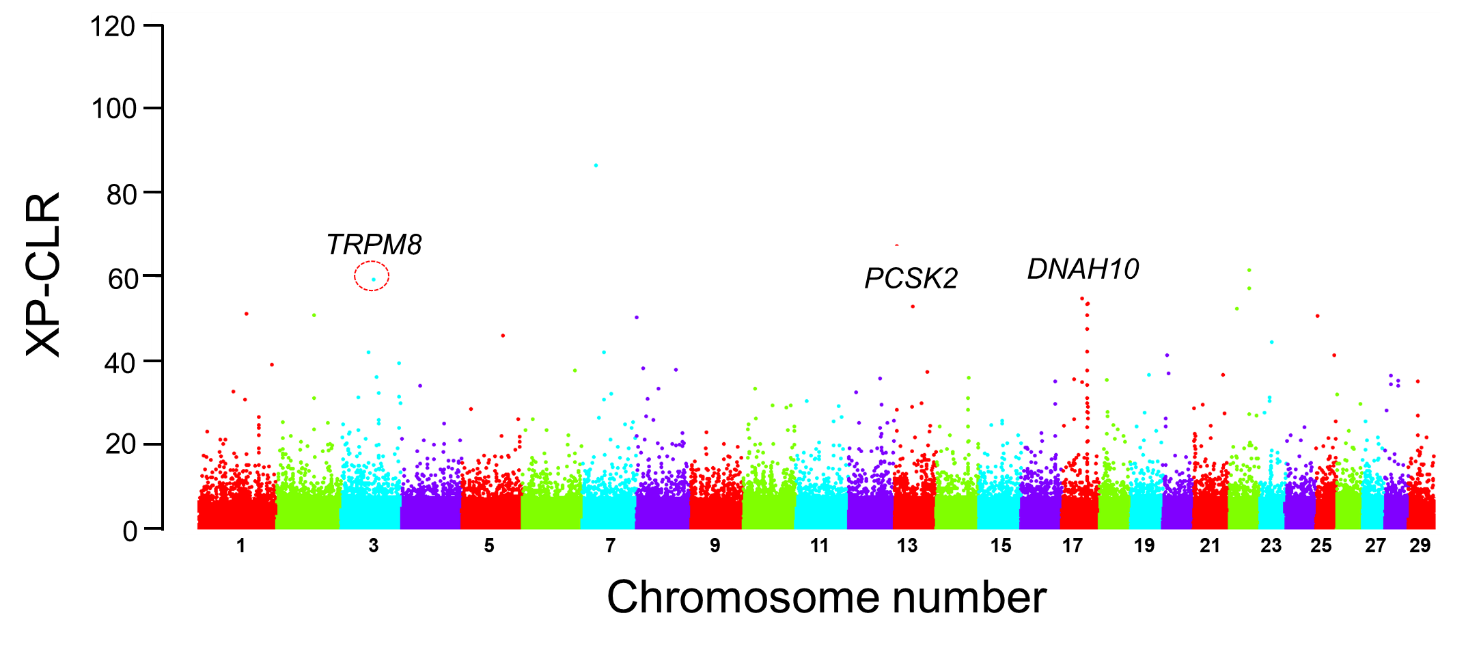

Supplement: Supplementary file 2 — Additional file 2 [file 12864_2023_9235_MOESM2_ESM.docx]
